# Temporal Dynamics of Sleep During Bright-Light Therapy for Depression and Their Relation to Symptom Improvement

**DOI:** 10.3390/clockssleep8020030

**Published:** 2026-05-26

**Authors:** Emma Visser, Niki Antypa, Machteld C. Marcelis, Claudia J. P. Simons, Yvonne A. W. de Kort

**Affiliations:** 1 Human Technology Interaction Group, Department of Industrial Engineering and Innovation Sciences, Eindhoven University of Technology, Den Dolech 2, 5612 AZ Eindhoven, The Netherlands; 2 Department of Clinical Psychology, Faculty of Social Sciences, Leiden University, Wassenaarseweg 2, 2333 AK Leiden, The Netherlands; nantypa@fsw.leidenuniv.nl; 3 Department of Psychiatry and Neuropsychology, Mental Health and Neuroscience Research Institute, Maastricht University, Vijverdalseweg 1, 6226 NB Maastricht, The Netherlands; machteld.marcelis@mumc.nl (M.C.M.); claudia.simons@ggze.nl (C.J.P.S.); 4 Department of Psychiatry and Psychology, Maastricht University Medical Center+ (MUMC+), P. Debyelaan 25, 6229 HX Maastricht, The Netherlands; 5 Mental Health Care Institute Eindhoven (GGzE), Dr. Poletlaan 40, 5626 ND Eindhoven, The Netherlands

**Keywords:** bright-lighttherapy, chronotherapy, major depressive disorder, circadian rhythms, sleep timing, sleep regularity, daily trajectories, longitudinal modelling, generalised additive mixed models

## Abstract

Sleep disturbance is a central feature of depression and a proposed pathway through which Bright-Light Therapy (BLT) exerts antidepressant effects. However, little is known about how sleep reorganises day by day during BLT or whether these dynamics relate to symptom improvement. We analysed daily sleep diaries from 66 patients with depression undergoing three weeks of BLT in routine outpatient care. Generalised Additive Mixed Models characterised daily trajectories in sleep timing, continuity, duration, and Subjective Sleep Quality, and weekly changes in sleep regularity were assessed using Root Mean Square of the Successive Differences. Structural Equation Modelling examined whether within-person deviations in sleep parameters mediated changes in depressive symptoms. Sleep timing showed gradual adjustment across treatment, with a progressive 48 min advance in weekday sleep onset. Sleep regularity improved from Week 1 to Week 2 before partially reversing, and the probability of nocturnal awakenings followed a non-linear trajectory. Other sleep parameters showed weaker directional trends. Improvements in Subjective Sleep Quality accounted for a modest portion of the association between treatment progression and reductions in depressive symptoms, whereas changes in sleep timing and regularity were not associated with symptom change. These findings indicate that sleep reorganises gradually during outpatient BLT, with different sleep dimensions evolving on distinct timescales and Subjective Sleep Quality emerging as one observable component linked to symptom improvement. More broadly, the results highlight the value of day-to-day modelling for understanding sleep–mood dynamics during real-world chronotherapy.

## 1. Introduction

Sleep disturbance is one of the most persistent and clinically burdensome symptoms of depressive disorders, yet the exact nature of these sleep disruptions differs greatly from one patient to another. The most frequently reported subjective sleep disturbances in depressed patients are insomnia, affecting up to 88% of individuals, and hypersomnia, present in roughly 27%, with these symptoms sometimes co-occurring and presenting as extended Sleep Onset Latency, frequent nocturnal awakenings, early morning awakenings, or increased daytime sleepiness [1,2,3]. These patterns matter clinically: prospective cohort studies show that insomnia symptoms and heightened night-to-night variability forecast the onset and persistence of depression and predict poorer treatment outcomes [4,5,6,7,8]. Moreover, experimental studies demonstrate that partial sleep deprivation and fragmented sleep increase emotional reactivity, impair prefrontal–limbic regulation, and heighten vulnerability to stress the following day [9,10,11]. Taken together, these findings underscore a robust bidirectional relationship in which the temporal organisation of sleep—its timing, continuity, and regularity—actively contributes to mood dysregulation [12,13].

The close relationship between sleep regulation and mood is particularly relevant in the context of Bright-Light Therapy (BLT), a frontline chronotherapeutic intervention for depressive disorders—including seasonal affective disorder, major depressive disorder, and bipolar depression—with rapid and robust clinical effects [14,15,16,17,18,19,20]. The therapeutic effects of BLT are believed to stem in part from its ability to reset and reinforce internal circadian rhythms: it shifts forward the frequently delayed circadian phase seen in patients with depression and may simultaneously enhance their typically reduced circadian amplitude [21,22,23,24]. These circadian pathways not only support mood regulation but also shape sleep timing, continuity, and day-to-day regularity, positioning sleep as one domain in which treatment-related changes may naturally emerge [22]. Consistent with this, clinical studies have reported earlier sleep timing, reduced fragmentation, and improvements in Subjective Sleep Quality following BLT [25].

But despite these findings, we know remarkably little about how sleep actually unfolds *during* BLT: whether changes appear immediately or gradually, whether different sleep dimensions follow shared or distinct temporal patterns, and how these evolving dynamics relate to the depressive symptom improvements. This gap largely reflects how BLT studies collect and analyse sleep. Often, sleep is only assessed at baseline and post-treatment or includes limited intermediate assessments, implicitly assuming that sleep improvements occur as relatively uniform shifts across the treatment period (e.g., [26,27,28,29]). Even when daily sleep diaries or continuous actigraphy measurements are collected, analyses typically rely on aggregated metrics that obscure night-to-night dynamics (e.g., [30,31,32]). Nonetheless, chronobiological theory and findings from other sleep-focused treatments such as cognitive behavioural therapy suggest that sleep responses may emerge non-linearly and at different rates across sleep domains, reinforcing the need to model sleep change at a fine temporal scale [33,34,35,36,37,38,39]. Collecting sleep data on a day-to-day basis would also allow us to examine sleep regularity—a core dimension of sleep health reflecting the stability of sleep timing across days—that has emerged as a key predictor of affective functioning but remains largely unexplored in BLT research [40].

Given the bidirectional link between sleep problems and depressive symptoms, understanding the temporal patterns in sleep during BLT is clinically important because it may provide insight into the mechanisms underlying BLT’s antidepressant effects. Even on a day-to-day level, diary and ecological momentary assessment studies consistently show that better sleep quality, longer and more consolidated sleep, and greater regularity predict improved next-day mood (often operationalised as affect) in both depressed and non-depressed samples [41]. In a 4-week open trial of morning BLT in depressed youth, early improvements in Subjective Sleep Quality and Sleep Onset Latency predicted subsequent reductions in depressive symptoms, suggesting that sleep improvements may act as an intermediate step in treatment response [42]. Complementing this, population-level actigraphy data indicate that associations between bright-light exposure and depressive symptoms may be statistically explained by improvements in sleep regularity [43]. Together, these findings support the hypothesis that sleep may contribute to the therapeutic effects of BLT. Yet direct longitudinal tests of such mediating pathways during BLT remain scarce.

The present study addresses these questions related to the temporal dynamics of sleep during BLT using daily sleep diaries collected over three weeks of BLT in routine clinical care. We use Generalised Additive Mixed Models to characterise daily trajectories in sleep timing, continuity, duration, and subjective quality, and we assess week-to-week shifts in night-to-night variability. Finally, we test whether these evolving sleep patterns—timing, variability, and subjective quality—mediate within-person changes in depressive symptoms. By combining temporal modelling with mediation analysis, the study provides a detailed account of how sleep changes during BLT and evaluates whether these dynamics associate with its antidepressant effects.

## 2. Results

### 2.1. Sample Description

The analytic sample included 66 adults (51.5% female; mean age = 38.5, *SD* = 13.9) with a current depressive episode in the context of major depressive disorder (60.6%), bipolar disorder (6.1%), and/or a seasonal affective pattern (37.9%) who completed at least three sleep diaries per week over three weeks of BLT. Sample descriptions are presented in Table 1. The participants reported moderate baseline severity of depressive symptoms (Quick Inventory of Depressive Symptomatology–Self-Report [QIDS-SR; [44]]: *M* = 16.7, *SD* = 4.07) and poor Subjective Sleep Quality in the month preceding data collection (Pittsburgh Sleep Quality Index [PSQI; [45]]: *M* = 9.4, *SD* = 3.48). Chronotype scores indicated an intermediate morningness–eveningness profile (Morningness–Eveningness Questionnaire [MEQ; [46]]: *M* = 43.5, *SD* = 12.4). Nearly half of the sample used antidepressant medication (45.5%) and 16.7% used sleep medication. A detailed overview of the specific medications used in the sample is provided in Supplementary Materials S1.

Among the participants with complete QIDS-SR data at baseline and Week 3 (*n* = 45), 17.8% met the remission criterion (QIDS-SR < 6) and 26.7% achieved a more-than-50% reduction in depressive symptoms.

### 2.2. Temporal Dynamics of Sleep Parameters During Bright-Light Therapy

#### 2.2.1. Generalised Additive Mixed Models

**Figure 1:**
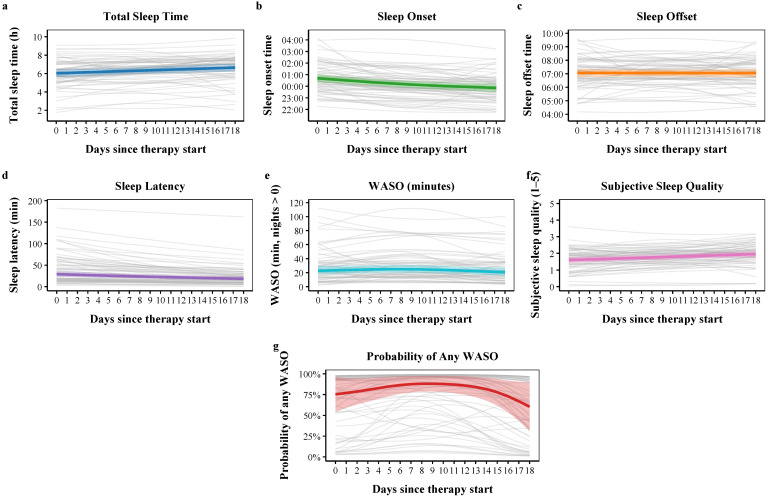
Population-level weekday smooths of sleep variables over time during Bright-Light Therapy, specifically (**a**) Total Sleep Time, (**b**) Sleep Onset, (**c**) Sleep Offset, (**d**) Sleep Latency, (**e**) Wake After Sleep Onset (WASO), (**f**) Subjective Sleep Quality, and (**g**) Probability of any WASO. Coloured lines indicate the population-level weekday smooths, with shaded area defining the 95% confidence interval. Grey lines depict participant-level weekday smooths.

The temporal trajectories of daily sleep parameters from the first Monday night of treatment until the last Thursday night were analysed using Generalised Additive Mixed Models (GAMMs) estimated with restricted maximum likelihood. Each model included a smooth term for therapy day and a participant-specific factor–smooth that allowed individuals to deviate from the group-level trajectory. Weekend was included both as a fixed effect and as weekend-specific smooths of therapy day to account for potential differences in sleep levels and temporal dynamics between weekdays and weekends. The models showed good overall fit, with adjusted $R^{2}$ values ranging from 0.48 to 0.70 and deviance explained between 49% and 75%. A complete overview of the model fit indices and smooth-term statistics is provided in Table 2, and population-level weekday trajectories are visualised in Figure 1. Standardised effect sizes are not reported for the temporal smooths, as these are inherently non-linear and their magnitude varies across time; instead, inference was based on smooth-term statistics and visualised trajectories with confidence intervals.

Across the three-week BLT period, weekday Sleep Onset advanced significantly, as indicated by an almost linear weekday smooth (s(Days):Weekday, effective degrees of freedom (edf) = 1.47, F=12.40, p<0.001, visualised in Figure 1b), corresponding to a 48 min advance from Day 0 (00:42) to Day 17 (23:54). In contrast, weekend Sleep Onset showed no evidence of temporal change (s(Days):Weekend, edf = 1.00, p=0.37), leading to a non-significant overall trend (s(Days), edf = 0.008, p=0.99).

The probability of experiencing any Wake After Sleep Onset (WASO) (Figure 1g) also changed significantly over time, reflected in a non-linear overall smooth (s(Days), edf = 1.75, $\chi^{2}=8.14$, p=0.020). The predicted probabilities declined modestly from 75 percent at Day 0 to approximately 67 percent at Day 17, with a shallow inverted-U trajectory: slightly increasing during early therapy, flat during mid-treatment, and decreasing toward the end. There was no evidence that weekday- or weekend-specific smooths differed significantly from the overall temporal pattern.

Subjective Sleep Quality (SSQ; Figure 1f) improved across therapy days, with significant temporal trends observed for both weekdays and weekends (weekday: s(Days):Weekday, edf = 1.00, F=9.67, p=0.002; weekend: edf = 1.00, F=4.45, p=0.035). The predicted values increased from approximately 1.60 to 1.93 on the 1–5 scale, corresponding to a gradual and largely linear improvement beginning at the start of therapy and continuing throughout the observation period.

In contrast, Total Sleep Time, Sleep Onset Latency (SOL), WASO duration, and Sleep Offset did not show significant population-level temporal effects (all p>0.05). Nonetheless, the predicted trajectories showed small directional tendencies: weekday Total Sleep Time increased by 35 min across the therapy period, SOL decreased by 10 min, WASO duration on nights with awakenings decreased by 11 min, and Sleep Offset shifted earlier by approximately 12 min.

Independent of temporal change, strong weekend effects were observed for several sleep characteristics: participants slept 1 h and 13 min longer on weekends (β=1.22 h, p<0.001), went to bed 17 min later (β=0.29 h, p=0.001), woke 1 h and 23 min later (β=1.39 h, p<0.001), showed slightly shorter SOL (β=−0.13 log-minutes, p=0.012), reported higher SSQ (β=0.23, p<0.001), and had a lower probability of WASO (β=−0.92, p<0.001), while WASO duration (if WASO occurred) did not differ significantly between weekdays and weekends (p=0.18).

Finally, participant-specific factor–smooths were significant for all outcomes (all p<0.001), indicating substantial between-person heterogeneity in both the intercept and the trajectory of the smooth terms. This heterogeneity is further illustrated by the wide range of participant-level smooths shown in Figure 1. To examine the potential sources of this variability, exploratory subgroup analyses are presented in Supplementary Materials S2.

#### 2.2.2. Effect of Confounder Adjustment on Temporal Smooths

To assess whether the baseline participant characteristics influenced inference regarding temporal sleep trajectories, we conducted sensitivity analyses in which each Generalised Additive Mixed Model was re-estimated including demographic, clinical, and circadian covariates (age, gender, baseline QIDS-SR, baseline PSQI, baseline MEQ, use of sleep medication, and use of antidepressant medication). The covariates were treated as baseline adjustment variables and were retained only if they improved the model fit based on the Akaike information criterion. Importantly, the smooth structure was kept identical to the primary models to ensure direct comparability of temporal trajectories. The full adjusted model results are reported in Supplementary Materials S3; the differences relative to the unadjusted models are indicated by symbols in Table 2.

Among the retained covariates, the baseline PSQI showed consistent associations with several sleep outcomes, including shorter Total Sleep Time (β=−0.20, p=0.001), earlier Sleep Offset (β=−0.13, p=0.008), longer SOL (β=0.14, p<0.001), greater WASO duration (β=0.08, p=0.012), and lower SSQ (β=−0.06, p=0.031). Age was positively associated with the probability of experiencing any WASO (β=0.08, p=0.043), and male gender was associated with shorter Total Sleep Time (β=−0.81, p=0.048). The baseline QIDS-SR was not significantly associated with any sleep outcome (all p>0.18). The baseline MEQ and the use of antidepressant or sleep medication were not retained in any of the models.

Including covariates did not materially change the general pattern of the predicted sleep trajectories compared with the primary models, although the statistical support for several temporal smooth terms differed. The progressive shift toward earlier Sleep Onset remained evident in the predicted trajectories, but covariate adjustment changed which day-type smooths were statistically supported, with a significant overall smooth and an additional weekend-specific effect emerging in the adjusted model (whereas the primary model showed day-type-specific support). Declines in SOL reached statistical significance only in the adjusted models; reductions in WASO probability shifted from an overall effect to a weekday-specific pattern, and subjective sleep-quality improvements were no longer statistically significant despite similar upward predicted trajectories.

### 2.3. Week-to-Week Variability in Sleep Timing and Duration

**Figure 2:**
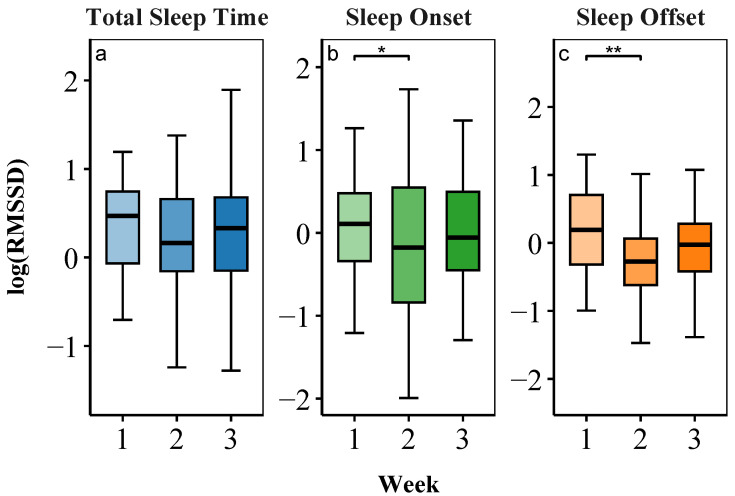
Weekly variability in sleep timing and duration during Bright-Light Therapy. Variability was quantified as the log-transformed Root Mean Square of the Successive Difference (log-RMSSD) for Total Sleep Time (**a**), Sleep Onset (**b**), and Sleep Offset (**c**). Boxplots show medians, interquartile ranges, and whiskers extending to 1.5× IQR. Asterisks indicate significant differences between weeks (* p<0.05, ** p<0.01).

To quantify week-to-week changes in night-to-night stability during BLT, we computed the Root Mean Square of the Successive Differences (RMSSD) for Total Sleep Time, Sleep Onset, and Sleep Offset for each participant and week (requiring at least three nights of data per week, after within-person outlier removal). The linear mixed-effects models were fitted to the log-transformed RMSSD values, with Week (1–3) as a fixed effect and random intercepts for the participants. Descriptive statistics for the RMSSD values on the original scale are provided in Table 3, and the distributions of log-RMSSD across weeks are shown in Figure 2.

Sleep Offset showed the clearest reduction in night-to-night variability across treatment. Variability decreased significantly over weeks (F(2,106.64)=6.17, p=0.003) with a marked decline from Week 1 to Week 2 (estimate =−0.41, SE=0.12, p=0.002). The contrast between Week 1 and Week 3 did not remain significant (p=0.064), indicating that the main change occurred early in treatment. On the original RMSSD scale, variability declined from 1.57 h in Week 1 to 1.12 h in Week 2 and remained similar in Week 3 (1.13 h), corresponding to an approximately 30% reduction between the first two weeks.

Sleep Onset variability showed a similar but smaller pattern. The effect of Week was significant, F(2,106.77)=3.98, p=0.021, again reflecting a decrease from Week 1 to Week 2 (estimate =−0.27, SE=0.10, p=0.019). Week 3 did not differ significantly from either Week 1 or Week 2. Mean RMSSD values declined from 1.38 h in Week 1 to 1.22 h in Week 2 before slightly increasing to 1.29 h in Week 3, corresponding to an approximate 12% reduction during the early treatment phase.

In contrast, variability in Total Sleep Time remained largely unchanged across the therapy period. The effect of Week was not significant (F(2,112.51)=1.35, p=0.26) and RMSSD values showed only small fluctuations (1.71 h, 1.46 h, and 1.62 h for Weeks 1–3).

Across the outcomes, the fixed effect of Week explained only a small proportion of the variance in night-to-night variability (marginal $R^{2}=0.01$–0.05), whereas the conditional $R^{2}$ values were substantially larger (0.33–0.55), indicating that most variability occurred at the individual level.

### 2.4. Mediation of Weekly Depressive Symptom Change by Weekly Sleep Parameters

**Figure 3:**
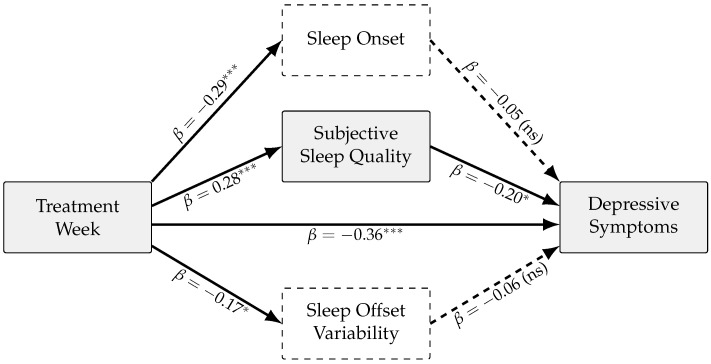
Path diagram of the parallel mediation model of weekly within-person associations between treatment week, sleep mediators, and depressive symptoms. Values on paths are standardised estimates (β; Std.all). Solid paths indicate statistically significant effects (* *p* < 0.05, *** *p* < 0.001), whereas dashed paths indicate non-significant effects (ns). Solid boxes indicate variables that are included in a significant mediation pathway. Dashed boxes are non-significant mediators. All variables were aggregated weekly and person-mean centred, such that estimates reflect within-person deviations. Depressive symptoms were assessed using the Quick Inventory of Depressive Symptoms, self-report with sleep items removed; Sleep Offset variability was operationalised as RMSSD of Sleep Offset times across the week.

We next examined whether within-person weekly deviations in selected sleep parameters statistically mediated within-person fluctuations in depressive symptoms during BLT using Structural Equation Modelling (SEM). Based on the temporal analyses and conceptual considerations, three complementary mediators were examined: mean Sleep Onset timing, RMSSD of Sleep Offset, and SSQ. SSQ was selected instead of the occurrence of awakenings because the latter was binary, strongly correlated with SSQ, and SSQ represents a broader dimension of subjective sleep disturbance commonly examined in the literature [41]. Weekly mediator values were aggregated from daily diaries and person-mean centred, such that the estimates reflected deviations from each participant’s typical level. Weekly depressive symptoms were assessed using the QIDS-SR (with sleep items removed) and also person-mean centred. The results of the mediation analysis are reported in Table 4 and visualised in Figure 3.

Across the treatment period, the later treatment weeks were associated with lower depressive symptom levels within individuals (c=−0.85, 95% CI [−1.17,−0.53], *p* < 0.001). This association remained significant after including the proposed mediators ($c^{\prime }=-0.78$, 95% CI [−1.14,−0.42], *p* < 0.001).

The tests of indirect effects showed one statistically significant mediation pathway. Treatment week exerted a significant indirect effect on depressive symptoms via higher Subjective Sleep Quality (SSQ) relative to an individual’s mean ($a_{3}b_{3}\;=\;-0.12$, bootstrapped 95% CI [−0.28,−0.02], *p* = 0.049), accounting for approximately 14% of the association between treatment week and depressive symptoms. The indirect pathways via earlier Sleep Onset relative to an individual’s mean ($a_{1}b_{1}\;=\;0.03$, bootstrapped 95% CI [−0.08,0.22], *p* = 0.662) and via sleep-offset variability ($a_{2}b_{2}\;=\;0.02$, bootstrapped 95% CI [−0.04,0.08], *p* = 0.441) were not significant.

Examining the component paths, later treatment weeks predicted earlier mean Sleep Onset within individuals ($a_{1}=-0.26$, 95% CI [−0.38,−0.14], *p* < 0.001), reduced variability in Sleep Offset ($a_{2}=-0.10$, 95% CI [−0.18,−0.01], *p* = 0.036), and higher Subjective Sleep Quality ($a_{3}=0.12$, 95% CI [0.06, 0.18], *p* < 0.001). When entered simultaneously in the outcome model, only SSQ was significantly associated with depressive symptoms ($b_{3}=-1.00$, 95% CI [−1.80,−0.19], *p* = 0.016), whereas mean Sleep Onset timing ($b_{1}=-0.11$, 95% CI [−0.59,0.37], *p* = 0.643) and Sleep Offset variability ($b_{2}=-0.21$, 95% CI [−0.72,0.30], *p* = 0.417) were not.

Because the model was saturated (df=0), global fit indices are not reported.

## 3. Discussion

The present study set out to characterise how sleep evolves on a day-to-day basis during outpatient BLT and whether these nightly dynamics co-vary with depressive symptom change within individuals. Whereas most prior BLT studies assess sleep only at baseline and post-treatment or rely on aggregated weekly metrics, the present modelling approach enables the examination of how multiple dimensions of sleep behaviour reorganise across the treatment window itself. By modelling daily sleep trajectories with Generalised Additive Mixed Models and separating within-person from between-person variation, this approach provides a more detailed characterisation of behavioural adaptation during treatment.

Overall, sleep behaviour showed gradual improvement over the treatment period, with most parameters changing in the expected direction but with varying magnitude and statistical support. The most pronounced changes were an advance in Sleep Onset timing, a reduction in nightly awakenings, and a decrease in sleep timing variability, particularly between the first and second week of treatment. Other characteristics—including Sleep Onset Latency, Subjective Sleep Quality, Total Sleep Time, Sleep Offset, and WASO duration—showed smaller or non-significant improvements after accounting for baseline sleep characteristics, depressive severity, and demographic factors. Within-person mediation analyses further indicated that improvements in sleep quality accounted for part of the reduction in depressive symptoms during therapy. Substantial inter-individual variability was observed both in sleep characteristics at the beginning of therapy and in the temporal trajectories over time.

Importantly, because the study was conducted in a structured outpatient setting (the LightCafé; [47]) and lacked a parallel control condition, these temporal changes cannot be attributed unequivocally to light exposure itself. Improvements in sleep timing and mood may partly reflect non-specific treatment effects, structured daily engagement, expectancy effects, behavioural activation, or the natural course of depressive episodes. Additionally, we did not quantify the actual light exposure received by the participants, nor did we control for environmental light exposure (e.g., seasonal variation in natural daylight). The present findings therefore provide a description of how sleep reorganises within an ecologically valid, real-world clinical setting. Accordingly, the observed effects should be interpreted as reflecting the combined influence of all components of the LightCafé setting rather than BLT in isolation.

### 3.1. Earlier Sleep Timing as the Most Coherent Behavioural Change During Treatment

A defining feature of the observed sleep dynamics is that most behavioural sleep parameters changed shortly after treatment initiation and evolved in a largely linear manner across the observation window. Within this pattern, the advance in Sleep Onset timing represents the most pronounced and behaviourally coherent change. This shift emerged early in treatment and was larger and more consistent than changes in other sleep dimensions, suggesting that sleep timing may be a particularly sensitive behavioural marker of treatment exposure in this specific treatment setting. Across the treatment window, weekday Sleep Onset advanced by approximately 48 min. This magnitude falls within the range of 30–60 min advances reported in prior BLT trials in depression and circadian sleep–wake disorders [30,48,49,50,51], and it is consistent with broader evidence showing that light therapy improves sleep timing in circadian-related sleep problems [25]. This advance may be clinically relevant given the frequent association between delayed sleep timing and depression (and the relatively late sleep timing observed in the present sample) [52,53], although the present data do not permit conclusions regarding underlying circadian alignment. Notably, these changes occurred within a stable weekly pattern, as weekends remained characterised by later sleep timing.

The advance in Sleep Onset may reflect an interplay between circadian, behavioural, and measurement-related influences. In principle, morning light exposure exerts a phase-advancing effect on the biological clock [54], which may promote earlier sleep initiation when schedules are not externally constrained [55,56]. At the same time, fixed early-morning attendance and increasing routine may reinforce earlier bedtimes independent of direct physiological phase adjustment [57,58,59]. Measurement characteristics may further accentuate this effect, as diary-reported Sleep Onset reflects a concrete behavioural decision that may be more salient and responsive to monitoring or expectancy effects than subtler aspects of nocturnal fragmentation [60,61,62]. The observed shift should therefore be interpreted primarily as behavioural realignment, as the present data do not allow firm conclusions about underlying circadian phase change.

### 3.2. Dynamic Changes in Sleep Regularity Across Treatment

Beyond the advance in sleep onset, sleep stability also changed during BLT. Variability in sleep timing—most clearly visible in sleep offset—declined between the first and second treatment week before partially increasing thereafter, indicating a dynamic rather than linear change in sleep regularity. Sleep regularity is a core dimension of sleep health, with greater night-to-night variability consistently associated with poorer mood regulation, higher depressive symptom severity, and reduced daytime functioning [40,63,64]. Consistent with this, behavioural sleep interventions such as cognitive behavioural therapy for insomnia and social rhythm therapy aim to stabilise sleep–wake timing, supporting the interpretation of reduced variability as favourable [57,65].

The reduction in variability may partly reflect the structured morning schedule imposed by daily light therapy sessions, requiring consistent wake times, particularly on weekdays. Such routines may directly constrain sleep timing and indirectly support circadian stabilisation by strengthening behavioural zeitgebers [57,58,66]. However, the lowest variability occurred during the second week, suggesting that scheduling alone does not fully explain the pattern. At the same time, similar reductions may reflect short-term behavioural compliance or treatment engagement. The subsequent partial rebound suggests that early regularisation did not continue to strengthen but instead evolved over time, possibly reflecting a shift from externally imposed routines toward more self-regulated schedules.

### 3.3. Non-Linear Reorganisation of Nocturnal Sleep Continuity

Nocturnal awakenings also followed a more irregular trajectory during treatment. The probability of awakenings changed non-linearly, with a transient increase early in treatment, stabilisation mid-treatment, and a decrease thereafter. Previous BLT studies have reported improvements in sleep continuity, often reflected in reduced WASO duration or improved sleep efficiency [25,30,67]. In the present data however, the only significant signal concerned the occurrence of awakenings rather than their duration. This difference may partly reflect the diary-based measurement approach, as recalling whether an awakening occurred is typically easier than estimating its duration [61,62]. These findings highlight that observed changes in sleep continuity depend on how it is operationalised and should be interpreted cautiously.

The early increase in awakenings may reflect a transient period of sleep instability during initial behavioural and circadian adjustment to the treatment. Because circadian phase advances in response to morning light unfold over several days rather than instantaneously [68], early treatment may involve a temporary mismatch between behavioural sleep timing and internal circadian phase, which can reduce sleep consolidation and efficiency [69]. Early changes in wake time and structured light exposure may therefore coincide with short-term instability in sleep continuity prior to broader circadian stabilisation. However, the present analyses do not allow conclusions about this sequencing, and the non-linear trajectory may also reflect contextual fluctuations or heterogeneous individual responses. Overall, these findings suggest that sleep continuity evolves more dynamically and less uniformly than sleep timing during treatment, underscoring the value of fine-grained temporal modelling.

### 3.4. Subjective Sleep Quality as a Partial Statistical Mediator of Symptom Improvement

The within-person mediation analysis provides insight into how behavioural sleep changes were statistically associated with clinical improvement during treatment. Improvements in Subjective Sleep Quality accounted for a modest portion of the association between later treatment weeks and lower depressive symptom levels (approximately 14%), whereas changes in Sleep Onset timing and Sleep Offset variability were not associated with symptom change. This suggests that sleep may represent one contributing pathway within the broader antidepressant response, consistent with previous findings linking sleep improvements to reductions in depressive symptoms across treatments, including cognitive behavioural therapy for insomnia [4,70]. However, the direct treatment–symptom association remained substantial, indicating that most symptom improvement cannot be explained by the measured sleep variables alone and may instead reflect additional mechanisms, including more direct mood-regulating effects of light exposure [71].

Importantly, sleep timing changes were not significantly associated with depressive symptom improvement, despite BLT generally being conceptualised as a chronotherapeutic intervention that exerts its antidepressant effects partly through the re-alignment of biological rhythms. However, an increasing number of studies have questioned the extent to which circadian phase shifts represent the main mechanism responsible for the antidepressant effects of BLT, and reported associations between circadian changes and clinical improvement have been mixed [72,73,74,75,76]. At the same time, because behavioural sleep timing only indirectly reflects underlying circadian physiology and because the present sample was relatively small and the mediation analysis had only limited temporal resolution the current findings do not permit strong conclusions regarding the importance of circadian mechanisms in BLT. Future studies incorporating direct physiological circadian markers are therefore needed to clarify the role of circadian re-alignment in treatment response.

One possible reason why Subjective Sleep Quality emerged as the only significant pathway is that, unlike individual timing or regularity metrics, it integrates multiple aspects of sleep, including duration, continuity, and depth, and may therefore better capture overall sleep improvement during BLT [77]. At the same time, Subjective Sleep Quality cannot be reduced to an aggregation of objective physiological parameters. Evidence from polysomnography and machine-learning studies shows that conventional sleep indices explain only a small proportion of variance in perceived sleep quality [78], suggesting it reflects a broader experiential construct beyond standard physiological metrics. As such, Subjective Sleep Quality may be particularly sensitive to changes in overall well-being, meaning that improvements may partly reflect broader positive changes rather than sleep processes alone. This may also explain why it is the sleep parameter most consistently associated with mood improvement in ecological momentary assessment and daily diary studies [41].

These results should be understood considering the specific set of of sleep variables incorporated into the mediation model, as well as the constraints of the analytical design. Sleep Onset timing, Sleep Offset variability, and Subjective Sleep Quality were selected because they showed clear longitudinal change, represented distinct dimensions of sleep, and exhibited limited multicollinearity. However, other sleep features, including Sleep Onset Latency, continuity measures, or multidimensional combinations of parameters, may also contribute to symptom improvement. Moreover, because the model estimates parallel weekly pathways using aggregated weekly data it cannot determine whether improvements in perceived sleep quality precede symptom change, follow symptom change, or reflect shared upstream processes. As such, the mediation analysis presented here should be interpreted as statistical rather than causal. In addition, because the main SEM was saturated, it is more informative about parameter estimates than about overall model fit. Future studies with finer temporal resolution and explicitly modelled sequential pathways may help clarify these relationships.

### 3.5. Methodological Considerations and Directions for Future Chronotherapy Research

Several methodological features shaped the inferences that can be drawn from the current results. Most importantly, the observed temporal sleep patterns were based on self-reported sleep rather than objective rest–activity measures or direct physiological circadian markers. Sleep diaries capture the experiential and behavioural organisation of sleep but are susceptible to recall bias and expectancy effects. Actigraphy, in contrast, provides movement-based estimates that are less influenced by subjective appraisal, yet may misclassify quiet wakefulness as sleep and overlook experiential aspects of consolidation. As these modalities capture different dimensions of sleep, subjective and objective indices do not necessarily evolve in parallel [30]. Future studies would benefit from combining actigraphy with daily diaries and incorporating direct circadian phase markers, such as dim-light melatonin onset or core body temperature rhythms, to determine whether observed behavioural changes in sleep timing correspond to shifts in objectively measured sleep and circadian phase over the course of treatment.

Several design features also affected interpretability and generalisability. The study was conducted in a structured outpatient setting that combined light exposure with routine reinforcement and behavioural guidance, meaning the observed sleep dynamics reflected adaptation within this context rather than the isolated physiological effects of light exposure alone and may have differed in less structured settings. At the same time, this naturalistic design enhances ecological validity and provides insight into how sleep reorganises during BLT in real-world clinical practice. The absence of a parallel control group and details on treatment adherence further precluded attribution of observed changes specifically to BLT and left open the possibility of non-specific treatment effects, environmental light influences, or natural symptom fluctuation.

Additional considerations relate to the analytic design. Only participants who completed three weeks of therapy and provided sufficient diary data were included, which may have under-represented early dropouts or rapid responders (although group comparisons showed no major differences between included and excluded participants on the available baseline characteristics). This also resulted in a relatively small sample size for both the GAMM and mediation analysis. Furthermore, the analyses focused on population-average trajectories and were not designed to capture individual response profiles, meaning distinct behavioural adjustment patterns may have remained undetected. Specifically, the presence of heterogeneous DSM-5 diagnoses and the associated use of different psychotropic medications may have differentially influenced sleep–wake regulation and treatment response. Indeed, supplementary visualisations suggest substantial between-person heterogeneity, with broadly similar overall trajectories but outcome-specific differences in levels and, to a lesser extent, patterns of change across subgroups. However, these observations were descriptive and based on limited subgroup sizes, and should therefore be interpreted with caution and investigated further in future research. Furthermore, sleep and mood were examined only during the active treatment phase, so the persistence of changes beyond therapy remains unknown. Finally, the retrospective structure of the diary (with entries completed on fixed days covering multiple preceding nights) may have smoothed day-to-day fluctuations, potentially reducing apparent variability in sleep timing and continuity.

## 4. Materials and Methods

### 4.1. Study Design

The current study is an exploratory secondary analysis of data from a previously completed observational trial conducted at the Mental Health Clinic Eindhoven and the Kempen (GGzE), the Netherlands [79]. The original trial evaluated the clinical impact of BLT delivered in a naturalistic café-like treatment setting across both summer and winter, with data collection taking place year-round between January 2021 and October 2022. As this study was observational in nature and involved participants receiving treatment as part of routine clinical care without experimental manipulation or prospective assignment to intervention conditions, prospective trial registration was not required by the institutional ethics board of the participating healthcare institute. To further enhance transparency, the study was nevertheless registered retrospectively on ClinicalTrials.gov (trial number: NCT07599124) in May 2026.

The present analysis focuses specifically on daily sleep diary measurements collected during treatment and examines day-to-day sleep patterns and their temporal association with changes in depressive symptoms. To this end, we restricted the dataset to participants receiving exactly three weeks of BLT, and we used only the the daily sleep diaries, repeated self-reported depressive symptom assessments, and baseline assessments. Analyses of the primary clinical outcomes of the effectiveness trial, focusing on overall changes in depressive symptoms, sleep quality and side effects, will be reported in the primary paper.

**Figure 4:**
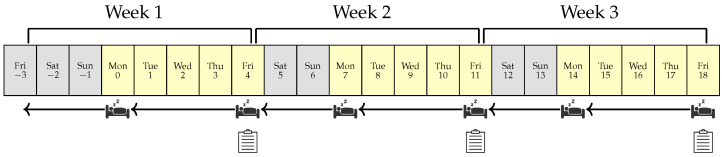
Study overview. Each box represents one calendar day (starting on a Friday). Yellow boxes indicate days on which participants received light therapy; grey boxes indicate days without therapy. Numbers inside the boxes denote days relative to treatment start (Monday = Day 0). Week labels indicate the aggregation windows used for week-to-week analyses. Sleep was recorded daily using sleep diaries; bed icons indicate the days on which sleep was retrospectively reported for the preceding nights. Notepad icons indicate assessment time points for depressive symptoms using the Quick Inventory of Depressive Symptomatology—Self Report (QIDS-SR).

A schematic overview of the relevant study timing and assessment structure is provided in Figure 4.

### 4.2. Participants

**Figure 5:**
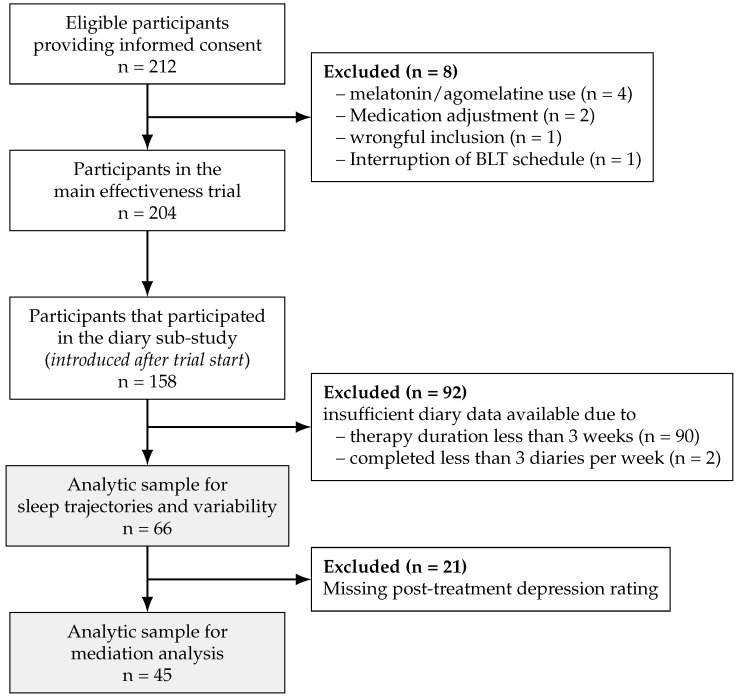
Flow diagram of participant inclusion for the diary sub-study and analytic samples. Grey boxes indicate final analytic samples.

In total, 212 participants met the inclusion criteria and provided written informed consent. Eight participants were subsequently excluded due to medication adjustment during the study (*n* = 2), use of melatonin or agomelatine (*n* = 4), wrongful inclusion (*n* = 1), or a vacation occurring between BLT weeks (*n* = 1), resulting in a full trial cohort of 204 participants. Of these, 158 participants participated in the sleep diary sub-study, which was introduced after the start of the main trial and therefore was not available for the earliest enrolled participants. Participants with fewer than three weeks of diary data were excluded (*n* = 92), leaving 66 participants for the analyses of sleep trajectories and night-to-night variability. For the mediation analysis, participants missing post-treatment depression scores were additionally excluded (*n* = 21), resulting in a final sample of 45. A flowchart detailing participant inclusion and exclusion is provided in Figure 5. No major differences were found between the included and excluded participants, as described in Supplementary Materials S4.

Eligibility was based on a physician-confirmed diagnosis of a current depressive episode, supported by a Quick Inventory of Depressive Symptomatology—Self-Report (QIDS-SR) score of six or higher [80]. Exclusion criteria included the presence of an active (hypo)manic or mixed episode, dementia, active suicidality, a current psychotic episode, changes in antidepressant medication within the three weeks preceding BLT, melatonin or agomelatine use in the month preceding BLT, or prolonged agomelatine use exceeding three weeks. Safety assessments for relative contraindications, including being in the first trimester of pregnancy, diabetes, light-sensitive eye conditions, epilepsy, antibiotic use, or the application of photosensitising creams, were conducted in consultation with the attending physician to ensure safe administration of BLT to each participant.

### 4.3. Intervention

BLT was implemented in accordance with the Dutch depression guidelines [81], utilising an Innolux incandescent BLT device ((Innosol Lucia 2 × 55 DIM, Innolux, Helsinki, Finland). The device emitted light with a 10,000 lux intensity at eye level and a correlated colour temperature of 3800 K. Treatment consisted of five 30 min sessions conducted on consecutive workdays (Monday to Friday) between 7:30 AM and 10:30 AM. The timing of the sessions was consistent across the study period and based on the preference and availability of the patient. Participants were free to engage in other activities, including the use of electronic devices like smartphones, during the treatment. Clinic staff monitored the proper positioning and recommended distance using a lux meter during the first session.

Therapeutic outcomes were assessed post-treatment on Friday using the QIDS-SR scale [80]. Participants achieving a score below 6 were considered to have a successful response and required no further therapy. Those with insufficient improvement (QIDS-SR score equal or higher than 6) received an additional week of therapy comprising five sessions. Treatment extensions were capped at two weeks, resulting in a potential duration of one to three weeks. Only participants that completed the full three weeks of therapy were included in the current analyses to ensure comparable sleep dynamics.

### 4.4. Setting

BLT was conducted at the LightCafé, a specialised outpatient care facility within GGzE, which is designed to provide BLT in conjunction with comprehensive lifestyle guidance within a restorative, non-stigmatising environment. In addition to receiving BLT, the participants engaged in daily semi-structured consultations with healthcare professionals, lasting 5 to 10 min. These consultations systematically addressed key domains of mental and physical well-being, including sleep hygiene, physical activity, nutritional habits, stress management, and the cultivation of meaningful social interactions. The goal was to equip the participants with practical strategies for improving both mental health and overall quality of life. The facility also provided tangible resources to support a healthy lifestyle. The participants had access to a range of nutritious options, such as fruits, tea, coffee, and water, as well as an exercise bike and opportunities to join organised walking groups. These components were strategically included to encourage active engagement in physical activity and health-promoting behaviours.

### 4.5. Measures

Sleep diaries were employed to assess the participants’ sleep–wake patterns during the treatment period. The diaries incorporated items from the Consensus Sleep Diary [82] to evaluate key variables, including bedtime, SOL, WASO, sleep offset, and SSQ. The participants completed the diaries in the clinic on Mondays and Fridays, reporting retrospectively on their sleep during the preceding nights (i.e., Friday–Sunday nights for Monday entries and Monday–Thursday nights for Friday entries).

Depressive symptoms were assessed every Friday using the Quick Inventory of Depressive Symptomatology-Self-Report (QIDS-SR; [80]). This validated instrument evaluated core domains of depression, such as mood, sleep, appetite, and cognitive function. The participants rated each of the 16 items based on their experiences over the past seven days using a 4-point Likert scale, with higher scores indicating greater symptom severity. The reliability and validity of the QIDS-SR have been well established in prior research [44]. For the mediation analysis, sleep items were removed from the total score of the QIDS-SR.

Additional baseline questionnaires assessed perceived sleep quality over the previous month using the Pittsburgh Sleep Quality Index (PSQI; [45]; scores > 5 indicate poor sleep quality) and chronotype using the Morningness–Eveningness Questionnaire (MEQ; [46]), with higher scores indicating greater morningness (16–41 evening type, 42–58 intermediate type, 59–86 morning type).

### 4.6. Statistical Analysis

All analyses were conducted in R (Version 4.5.0).

Daily sleep variables were derived from sleep diaries and included Sleep Onset, Sleep Offset, SOL, WASO, SSQ, and Total Sleep Time (TST). Sleep Onset was calculated as bedtime plus SOL. TST was calculated as the time from Sleep Onset to Sleep Offset, minus WASO. Daily entries with implausible values (e.g., TST ≤ 0 h) or participant-specific outliers exceeding ±3 SD were set to missing (less than 2% of entries). To account for zero inflation, WASO was decomposed into two components: the occurrence of WASO on a given night (yes/no) and, when WASO occurred, its duration (minutes). SOL and WASO duration were right-skewed and analysed on the log(x+1) scale. Sleep Onset times crossing midnight were converted to a continuous “across-midnight” scale so that later Sleep Onset (e.g., 01:00 h) was coded as 25. Missing diary entries were not imputed. All longitudinal models were therefore estimated from the available observations after preprocessing and outlier removal.

#### 4.6.1. Temporal Dynamics of Daily Sleep (GAMMs)

To examine temporal trajectories in daily sleep, we fitted Generalised Additive Mixed Models (GAMMs) using mgcv with restricted maximum likelihood (REML). For each continuous outcome (TST, sleep onset, sleep offset, log-SOL, log-WASO duration, SSQ) the models included a parametric weekend-versus-weekday effect to capture average level differences, a population-level smooth for therapy day, a day-type-specific smooth deviation allowing weekday and weekend trajectories to differ over time, and a participant-specific factor-smooth for therapy day. WASO occurrence was analysed analogously using a binomial GAMM with a logit link. The analyses were conducted on diary data from the first night after treatment start through the final recorded night at the end of treatment. Friday and Saturday night were considered the weekend.

Basis dimensions were intentionally kept modest (k = 6 for the overall smooth, k = 3 for the weekend-specific smooth, and k = 3 for the participant-specific factor-smooth) because the treatment period comprised only approximately three weeks of diary observations. This choice was made to allow non-linearity while avoiding overfitting. Smoothness was estimated by REML, such that the effective degrees of freedom were selected by penalisation rather than fixed a priori, and model adequacy was checked using gam.check. Full model formulas, smoothing specifications, and diagnostics are presented in Supplementary Materials S5.

To complement the significance tests, we summarised the primary temporal trends using model-based predicted change across treatment and corresponding 95% confidence intervals on the response scale. We do not report standardised effect sizes for the temporal smooths because their inherently non-linear form means their magnitude varies across time.

To assess whether baseline differences could account for the observed temporal patterns, we refitted each model including a set of additive covariates as potential confounders: age, gender, baseline depressive symptom severity (QIDS-SR), baseline Subjective Sleep Quality (PSQI), chronotype (MEQ score), sleep-medication use, and antidepressant use. The confounders were evaluated using a forward stepwise procedure based on the Akaike Information Criterion (AIC): each covariate was added sequentially and retained only if it reduced the AIC by more than two points, indicating meaningful improvement in model fit. Smooth terms and weekend/weekday contrasts were otherwise kept identical to the base models to ensure direct comparability.

#### 4.6.2. Weekly Sleep Variability (RMSSD)

To examine week-to-week variability in sleep timing and duration, we computed the Root Mean Square of the Successive Differences (RMSSD) for Sleep Onset, Sleep Offset, TST, SOL, and WASO using daily values ordered within participant and week. RMSSD was calculated only for participant-weeks with at least three observed nights, and missing diary days were not imputed. Participant-weeks with fewer than three observed nights were excluded from RMSSD estimation. For each RMSSD outcome, a linear mixed-effects model was fitted with week as a categorical fixed effect and participant as a random intercept. Pairwise week contrasts were estimated from these models with Bonferroni adjustment.

#### 4.6.3. Weekly Mediation of Depressive Symptoms

To examine whether changes in sleep were statistically associated with improvements in depressive symptoms during BLT, a within-person parallel mediation analysis was conducted using weekly participant-level data.

Sleep variables were selected a priori based on the preceding longitudinal GAMM and variability analyses. The measures showing the most pronounced longitudinal change were mean Sleep Onset time, Sleep Offset variability, SOL, SSQ, and the probability of WASO. Because SOL and WASO were strongly correlated with SSQ, and SSQ is the most widely used daily indicator of sleep in studies of sleep–mood associations [41], we retained SSQ to represent this domain. To avoid multicollinearity, the final mediation model therefore included mean Sleep Onset time, Sleep Offset variability, and SSQ, capturing distinct aspects of sleep timing, variability, and subjective quality. The outcome consisted of weekly QIDS-SR scores with sleep-related items removed. All mediators and the outcome were person-mean centred so that coefficients reflected within-person deviations across the three therapy weeks. Treatment week was coded 1–3 and centred within participant.

Parallel mediation was tested using a single-level Structural Equation Model implemented in lavaan. Each mediator was regressed on centred treatment week (the *a*-paths), and weekly person-centred QIDS-SR scores were regressed simultaneously on treatment week (direct effect $c^{\prime }$) and all mediators (the *b*-paths). Residual covariances among mediators were freely estimated. Models were estimated using robust maximum likelihood with full-information maximum likelihood to handle missing observations. This yielded robust standard errors and robust Wald confidence intervals for the *a*-paths, *b*-paths, direct effect, and total effect. Indirect effects were defined as the product of the corresponding *a*- and *b*-paths and were evaluated using percentile bootstrap confidence intervals based on 5000 resamples. The model was saturated (df=0), and therefore global fit indices were not interpreted.

## 5. Conclusions

The present study provides a temporally detailed behavioural account of how sleep reorganises during routine outpatient BLT. Rather than changing uniformly, different dimensions of sleep followed distinct developmental courses across the treatment window: sleep timing advanced early and progressively, sleep-offset variability showed a transient decrease before partially increasing again, and nocturnal awakenings evolved dynamically with non-linear adjustment. Within individuals, weeks characterised by relatively better perceived sleep quality tended also to coincide with lower depressive symptom burden, suggesting that improvements in sleep represent one observable component within the broader therapeutic response. Taken together, these findings suggest that this specific BLT setting does not simply trigger one specific, isolated change in sleep. Instead, it appears to set in motion a progressive reorganisation of everyday behavioural rhythms across multiple timescales, which in turn is associated with its antidepressant effect.

In general, the present study should be understood as a behavioural description of how sleep is reorganised during outpatient BLT, rather than as a conclusive examination of the temporal dynamics of sleep or the specific physiological mechanisms involved. Despite its methodological limitations, the study’s focus on day-to-day dynamics, the within-person analytic approach, and real-world clinical context provide a uniquely detailed behavioural account of sleep reorganisation during BLT, on which future work can build by integrating controlled designs, physiological circadian markers, objective ambulatory monitoring, and extended follow-up assessments to more precisely disentangle circadian, behavioural, and contextual processes in treatment response.

## Figures and Tables

**Table 1:** Sample characteristics.

Variable	Mean (SD) or N (%)
**Demographics**	
Age (years)	38.5 (13.92)
Gender (female)	34 (51.5%)
**Clinical characteristics**	
Baseline QIDS-SR	16.7 (4.07)
Baseline PSQI	9.4 (3.48)
**DSM-5 diagnoses**	
Depressive Disorder	40 (60.6%)
Seasonal Affective Disorder †	25 (37.9%)
Bipolar Disorder	4 (6.1%)
Dysthymia	5 (7.6%)
Autism Spectrum Disorder	16 (24.2%)
Personality Disorder	13 (19.7%)
ADHD	12 (18.2%)
PTSD	7 (10.6%)
**Medication**	
Antidepressant use	30 (45.5%)
Sleep medication use	11 (16.7%)
**Circadian traits**	
Chronotype (MEQ total score)	43.5 (12.43)
**Treatment response**	
Remission (QIDS-SR < 6)	8 (17.8%)
Response (50% symptom reduction)	12 (26.7%)
Mean point reduction	4.40 (4.35)

*Note*. Total sample consisted of 66 participants, but only 45 completed both baseline and end-of-treatment depression ratings. QIDS-SR = Quick Inventory of Depressive Symptomatology, Self-Report; PSQI = Pittsburgh Sleep Quality Index; ADHD = Attention-deficit/hyperactivity disorder; PTSD = Post-Traumatic Stress Disorder; MEQ = Morningness–Eveningness Questionnaire. Participants could meet criteria for multiple DSM-5 diagnoses. † Seasonal Affective Disorder was defined as meeting at least three of the four DSM-derived seasonal pattern criteria: recurrent winter episodes, remission outside of winter, a pattern persisting for at least two years, and a higher frequency of winter than summer episodes. Boldface is used to indicate grouped variable headings.

**Table 2:** Model fit indices, fixed effects, and smooth-term statistics From GAMMs examining temporal dynamics of sleep during Bright-Light Therapy.

Outcome	*n*	EDF_total_	Adj. $R^{2}$	Dev. Expl. (%)	AIC	Fixed Effect (Weekend)	Smooth Term	edf	Test Stat	*p*
Total Sleep Time (hours)	959	125	0.61	65.8	3305	β=1.22, t=11.40, p<0.001	s(Days)	1.00	F=0.45	0.505
							s(Days):Weekday	1.00	F=0.32	0.570
							s(Days):Weekend	0.0001	F=0.07	0.997
							s(Days,subjno)	123	F=6.65	<0.001
Sleep onset	954	127	0.61	66.0	2856	β=0.29, t=3.30, p=0.001	s(Days)	0.008	F=0.00	${0.995\;}^{§}$
							s(Days):Weekday	1.47	F=12.40	<${0.001\;}^{†}$
							s(Days):Weekend	1.00	F=0.81	${0.369\;}^{§}$
							s(Days,subjno)	125	F=7.21	<0.001
Sleep offset	949	140	0.72	76.1	2235	β=1.39, t=22.10, p<0.001	s(Days)	1.00	F=3.37	0.067
							s(Days):Weekday	1.00	F=3.66	0.056
							s(Days):Weekend	0.0002	F=0.12	0.995
							s(Days,subjno)	138	F=9.67	<0.001
Sleep latency (log min +1)	955	102	0.65	68.7	1903	β=−0.13, t=−2.51, p=0.012	s(Days)	1.00	F=1.76	${0.184\;}^{§}$
							s(Days):Weekday	1.00	F=0.27	0.601
							s(Days):Weekend	0.0002	F=0.01	0.998
							s(Days,subjno)	99.9	F=8.84	<0.001
WASO (log min +1; nights > 0)	619	107	0.66	71.9	1178	β=0.09, t=1.34, p=0.18	s(Days)	1.00	F=0.56	0.454
							s(Days):Weekday	1.63	F=0.67	0.405
							s(Days):Weekend	0.0001	F=0.26	0.993
							s(Days,subjno)	104	F=6.76	<0.001
Pr(any WASO) ^*a*^	933	97.8	0.59	57.7	712	β=−0.92, z=−3.33, p<0.001	s(Days)	1.75	$\chi^{2}=8.14$	${0.020\;}^{†}$
							s(Days):Weekday	1.00	$\chi^{2}=0.97$	${0.325\;}^{§}$
							s(Days):Weekend	1.00	$\chi^{2}=1.36$	0.244
							s(Days,subjno)	94.1	$\chi^{2}=320.31$	<0.001
Subjective Sleep Quality (1–5)	941	113	0.48	54.0	2080	β=0.23, t=4.00, p<0.001	s(Days)	0.003	F=0.23	0.975
							s(Days):Weekday	1.00	F=9.67	${0.002\;}^{†}$
							s(Days):Weekend	1.00	F=4.45	${0.035\;}^{†}$
							s(Days,subjno)	111	F=4.24	<0.001

*Note.* EDF = effective degrees of freedom. WASO = Wake After Sleep Onset. ^*a*^ Binomial GAMM with logit link; smooths evaluated using $\chi^{2}$ tests. ^†^ Significant in this unadjusted model but not in the confounder adjusted model; ^§^ not significant in this unadjusted model but significant after confounder adjustment.

**Table 3:** Weekly RMSSD, omnibus tests, and pairwise contrasts for sleep variability.

Outcome	Week 1	Week 2	Week 3	Omnibus	Contrast	*b*	SE	*p*
Total Sleep Time	1.71 (1.34)	1.46 (0.89)	1.62 (1.14)	F(2,112.5)=1.35,	2–1	−0.17	0.10	0.314
				p=0.264	3–1	−0.08	0.10	1.00
					3–2	0.09	0.10	1.00
Sleep Onset	1.38 (1.04)	1.22 (1.05)	1.29 (0.92)	F(2,106.8)=3.98,	2–1	−0.27	0.10	0.019
				p=0.021	3–1	−0.12	0.10	0.722
					3–2	0.16	0.09	0.280
Sleep Offset	1.57 (1.07)	1.12 (1.02)	1.13 (0.97)	F(2,106.6)=6.17,	2–1	−0.41	0.12	0.002
				p=0.003	3–1	−0.27	0.12	0.064
					3–2	0.14	0.11	0.645

*Note*. RMSSD = Root Mean Square of the Successive Differences. Values in parentheses are standard deviations on the original scale. Omnibus tests are Type III tests of the fixed effect of week from linear mixed-effects models. Pairwise contrasts (*b*, SE) are based on models fitted to log-transformed RMSSD values with random intercepts for participants; *p* values are Bonferroni adjusted.

**Table 4:** Mediation analysis using Structural Equation Modelling linking treatment week, sleep parameters, and depressive symptoms.

Path	Predictor → Outcome	*B*	SE	β	95% CI ^**†**^	*p*
**a-paths (Week → Mediators)**						
$a_{1}$	Week → Onset	−0.26	0.06	−0.29	[−0.38, −0.14]	<0.001
$a_{2}$	Week → Offset var.	−0.10	0.05	−0.17	[−0.18, −0.01]	0.036
$a_{3}$	Week → Quality	0.12	0.03	0.28	[0.06, 0.18]	<0.001
**b-paths (Mediators → Dep. Symp.)**						
$b_{1}$	Onset → Dep. symp.	−0.11	0.25	−0.05	[−0.59, 0.37]	0.643
$b_{2}$	Offset var. → Dep. symp.	−0.21	0.26	−0.06	[−0.72, 0.30]	0.417
$b_{3}$	Quality → Dep. symp.	−1.00	0.41	−0.20	[−1.80, −0.19]	0.016
**Direct effect**						
$c^{\prime }$	Week → Dep. symp.	−0.78	0.19	−0.36	[−1.14, −0.42]	<0.001
**Indirect effects**						
	Week → Onset → Dep. symp.	0.03	0.07	0.01	[−0.08, 0.22]	0.662
	Week → Offset var. → Dep. symp.	0.02	0.03	0.01	[−0.04, 0.08]	0.441
	Week → Quality → Dep. symp.	−0.12	0.06	−0.06	[−0.28, −0.02]	0.049
**Total effect**						
	Week → Dep. symp. (total)	−0.85	0.17	−0.39	[−1.17, −0.53]	<0.001

*Note.B* = unstandardised estimate; β = standardised estimate (Std.all). All variables were aggregated weekly and person-mean centred, such that estimates reflect within-person deviations. Onset = mean Sleep Onset timing; Offset var. = RMSSD sleep-offset; Quality = Subjective Sleep Quality; Dep. sympt. = depressive symptom severity (assessed using the Quick Inventory of Depressive Symptomatology, Self-Report; sleep items removed). The model was saturated (df=0), therefore global fit indices are not informative and are not reported. † Confidence intervals for the a-paths, b-paths, direct effect, and total effect are robust Wald confidence intervals obtained with the MLR estimator. Confidence intervals for the indirect effects are bootstrap confidence intervals based on 5000 resamples. Boldface is used to distinguish different sets of paths in the mediation model.

## Data Availability

The data supporting the findings of this study are not publicly available due to privacy restrictions and the conditions of participant consent.

## Supplementary Materials

The following supporting information can be downloaded at: https://www.mdpi.com/article/10.3390/clockssleep8020030/s1. Section S1: Specific Medications Used in the Sample, including Table S1: Psychotropic and sleep-related medications used in the sample. Section S2: Individual Heterogeneity and Subgroup Visualizations, including Figure S1: Exploratory subgroup-specific population-level weekday trajectories of sleep variables over time during bright-light therapy, stratified by baseline Pittsburgh Sleep Quality Index (PSQI) scores; Figure S2: Exploratory subgroup-specific population-level weekday trajectories of sleep variables over time during bright-light therapy, stratified by treatment response status (responders vs. non-responders); Figure S3: Exploratory subgroup-specific population-level weekday trajectories of sleep variables over time during bright-light therapy, stratified by chronotype; and Figure S4: Exploratory subgroup-specific population-level weekday trajectories of sleep variables over time during bright-light therapy, stratified by depression diagnosis (unipolar vs. bipolar). Section S3: Confounder-Adjusted GAMMs, including Table S2: Model fit indices, fixed effects, and smooth-term statistics from confounder-adjusted GAMMs examining temporal dynamics of sleep during bright-light therapy. Section S4: Included vs. Excluded Participants, including Table S3: Comparison of participants included in versus excluded from the sleep-analysis sample. Section S5: GAMM Specification and Diagnostic Checks, including Table S4: GAMM convergence and fit diagnostics; and Table S5: Basis-dimension diagnostics from gam.check().

## Abbreviations

The following abbreviations are used in this manuscript:

BLT	Bright-Light Therapy
GAMM	Generalised Additive Mixed Model
MEQ	Morningness–Eveningness Questionnaire
PSQI	Pittsburgh Sleep Quality Index
QIDS-SR	Quick Inventory of Depressive Symptomatology, Self-Report
RMSSD	Root Mean Square of the Successive Differences
SEM	Structural Equation Modelling
SOL	Sleep Onset Latency
SSQ	Subjective Sleep Quality
TST	Total Sleep Time
WASO	Wake After Sleep Onset
