# Supplementary material for: Temporal Dynamics of Sleep During Bright-Light Therapy for Depression and Their Relation to Symptom Improvement
Source: Clocks Sleep. 2026 May 26;8(2):30. doi: 10.3390/clockssleep8020030 (PMC13298590; doi:10.3390/clockssleep8020030)
Supplement: Supplementary file 1 [file clockssleep-08-00030-s001.zip › clockssleep-4238622-supplementary.pdf]

Supplementary Materials:

S1. Specific Medications Used in the Sample

Table S1. Psychotropic and sleep-related medications used in the sample

	**N (%)**	**mg/day** median (range)
**SSRI antidepressant**		
Escitalopram	5 (7.6%)	20 (10-20)
Fluoxetine	3 (4.5%)	60 (40-60)
Sertraline	3 (4.5%)	75 (50-100)
Citalopram	1 (1.5%)	20
Fluvoxamine	1 (1.5%)	50
Paroxetine	1 (1.5%)	20
**TCA antidepressant**		
Nortriptyline	5 (7.6%)	75 (50-150)
Amitriptyline	1 (1.5%)	25
Clomipramine	1 (1.5%)	75
**Other antidepressant**		
Bupropion	6 (9.1%)	150 (150-300)
Mirtazapine	5 (7.6%)	22.5 (7.5-30)
Duloxetine	1 (1.5%)	120
Trazodon	1 (1.5%)	100
**Benzodiazepines and related hypnotics**		
Oxazepam	5 (7.6%)	20 (20-20)
Diazepam	3 (4.5%)	2 (2-5)
Temazepam	2 (3 %)	20 (20-20)
Zolpidem	2 (3 %)	–
Alprazolam	1 (1.5%)	0.2
Clobazam	1 (1.5%)	20
Midazolam	1 (1.5%)	7.5
**Phenothiazine antihistamine**		
Promethazine	4 (6.1%)	37.5 (25-50)
**Antipsychotics**		
Quetiapine	7 (10.6%)	25 (6.2-100)
Aripiprazol	5 (7.6%)	9.5 (2.5-15)
Risperidon	3 (4.5%)	1.5 (0.5-2)
Pipamperon	1 (1.5%)	100
**Mood stabilizers and anticonvulsants**		
Topiramaat	4 (6.1%)	175 (25-250)
Lamotrigine	2 (3 %)	200 (100-300)
Lithium	1 (1.5%)	500
Valproinezuur	1 (1.5%)	1250
Carbamazepine	1 (1.5%)	1000
**Other psychotropic medications**		
Pregabaline	2 (3 %)	300 (150-450)
CBD olie	1 (1.5%)	–

Note. Percentages are based on the total sample of 66 participants. Participants could use more than one medication and could therefore contribute to multiple rows. Dose/day summaries are drug-specific and were calculated only when a numeric daily frequency could be derived; non-numeric schedules or unclear entries were left blank. Medications are grouped by their formal pharmacological class rather than by the clinical indication for which they may have been prescribed in this sample. Medication use in the sample was heterogeneous, including several antidepressant classes as well as benzodiazepines, hypnotics, antipsychotics, mood-stabilizing agents, and other psychotropic medications. Because the likely effects of these medications on sleep may differ across specific drugs, dosages, timing of administration, treatment duration, and sleep outcomes, this level of pharmacological detail was not incorporated into the main longitudinal models.

S2. Individual Heterogeneity and Subgroup Visualizations

To examine whether sample-average trajectories obscured meaningful heterogeneity, we generated subgroup-specific trajectory visualizations for treatment response status (yes/no), baseline PSQI quartiles (upper and lower 25%), baseline MEQ quartiles (upper and lower 25%), and diagnosis (unipolar/bipolar). This was done by re-fitting the same GAMM specification separately within each subgroup and visually comparing the resulting trajectories, rather than by including subgroup interaction terms in the primary model. This approach was chosen because adding multiple interaction terms to already complex GAMMs would substantially increase model complexity and result in convergence issues.

Subgroup-specific trajectories were estimated for total sleep time, sleep onset, sleep offset, sleep latency, WASO duration, and subjective sleep quality. The probability of any WASO was also explored, but these estimates appeared unstable across subgroups and were therefore omitted from the final analysis. For each model, predicted population-level trajectories were extracted across the observed range of days since therapy start and displayed as fitted lines with 95% uncertainty bands.

These visualizations were intended as descriptive only and were not evaluated as formal subgroup analyses. In line with this, we did not report subgroup-specific smooth-term statistics, as the limited subgroup sizes would yield unstable and potentially misleading estimates. Instead, visual inspection was used to provide a qualitative overview of potential heterogeneity in trajectories. Apparent between-group differences should therefore be interpreted as hypothesis-generating rather than conclusive.

Across subgroup visualizations, temporal patterns closely followed those observed in the full sample, with changes emerging early in treatment and evolving gradually thereafter. Within this overall consistency, however, small but potentially meaningful differences between subgroups became visible, depending on the outcome (Figures S1–S4).

The most pronounced contrasts were observed across baseline PSQI quartiles (Figure S1). Participants with poorer baseline sleep consistently showed less favourable levels across outcomes. For certain parameters—particularly sleep latency and WASO duration—this group also appeared to show more marked, non-linear improvements over time, whereas for outcomes such as total sleep time and subjective sleep quality, differences were mainly reflected in overall levels rather than in the pattern of change. When comparing responders with non-responders (Figure S2), distinctions were again primarily visible in overall levels, with responders showing more favourable sleep profiles. There were also modest indications of slightly different rates of improvement among responders for some measures, especially sleep onset and latency. In contrast, trajectories across MEQ quartiles were largely similar across subgroups despite clear baseline differences in sleep timing (Figure S3). Diagnosis-specific plots hinted at potential differences (Figure S4), but wide uncertainty bands—particularly in the bipolar subgroup—made these patterns difficult to interpret.

Taken together, these visualizations indicate substantial heterogeneity in the temporal dynamics of sleep during BLT. However, the specific patterns should be interpreted with caution given the limited subgroup sizes. Future research is needed to examine these potential subgroup differences more systematically.

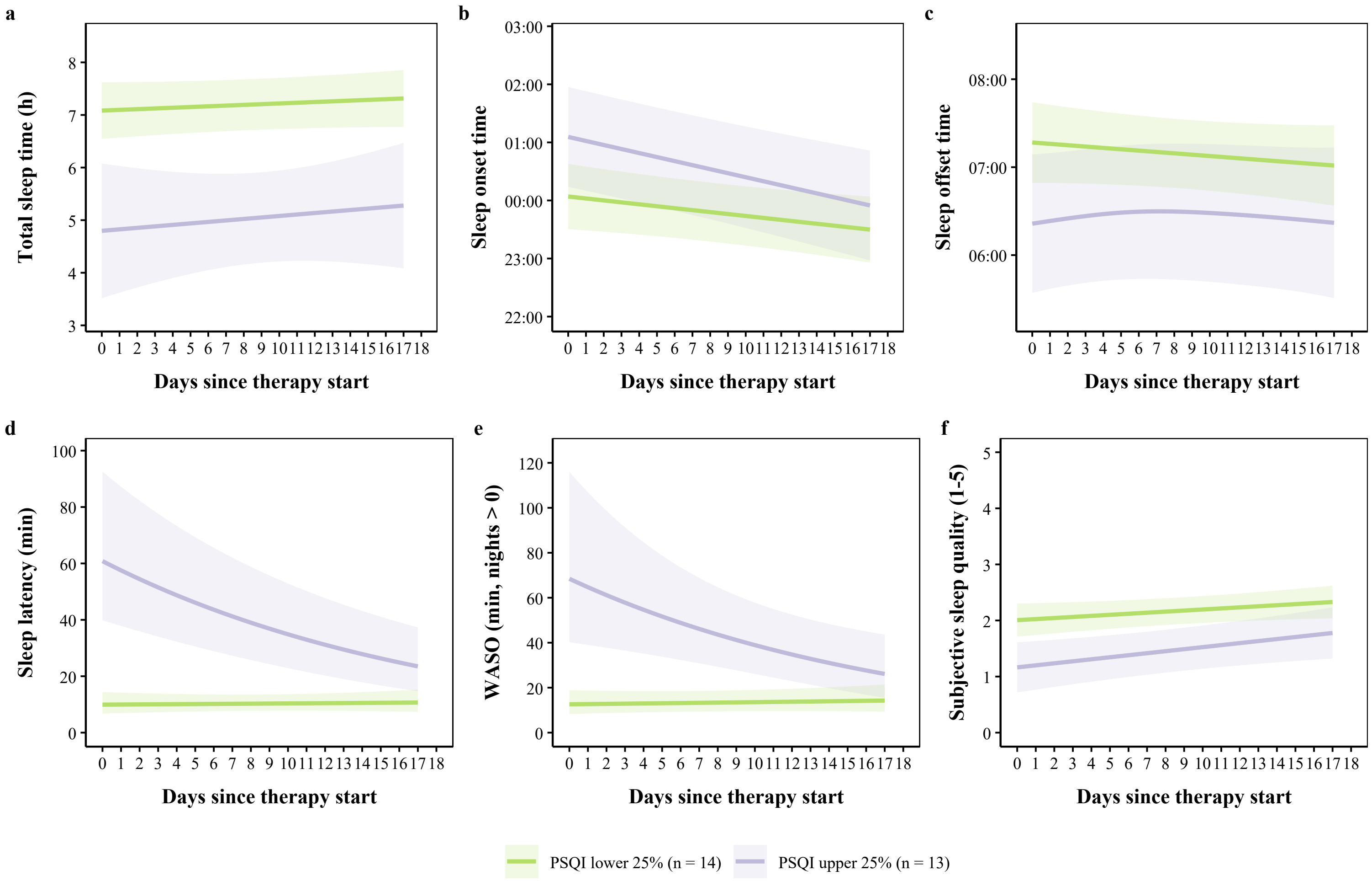

Figure S1. Exploratory subgroup-specific population-level weekday trajectories of sleep variables over time during bright-light therapy, stratified by baseline Pittsburgh Sleep Quality Index (PSQI) scores. Subplots show (a) total sleep time, (b) sleep onset time, (c) sleep offset time, (d) sleep latency, (e) Wake After Sleep Onset (WASO), and (f) subjective sleep quality. Curves were obtained by re-fitting the main GAMM separately within each subgroup and are shown with uncertainty bands. WASO = wake after sleep onset.

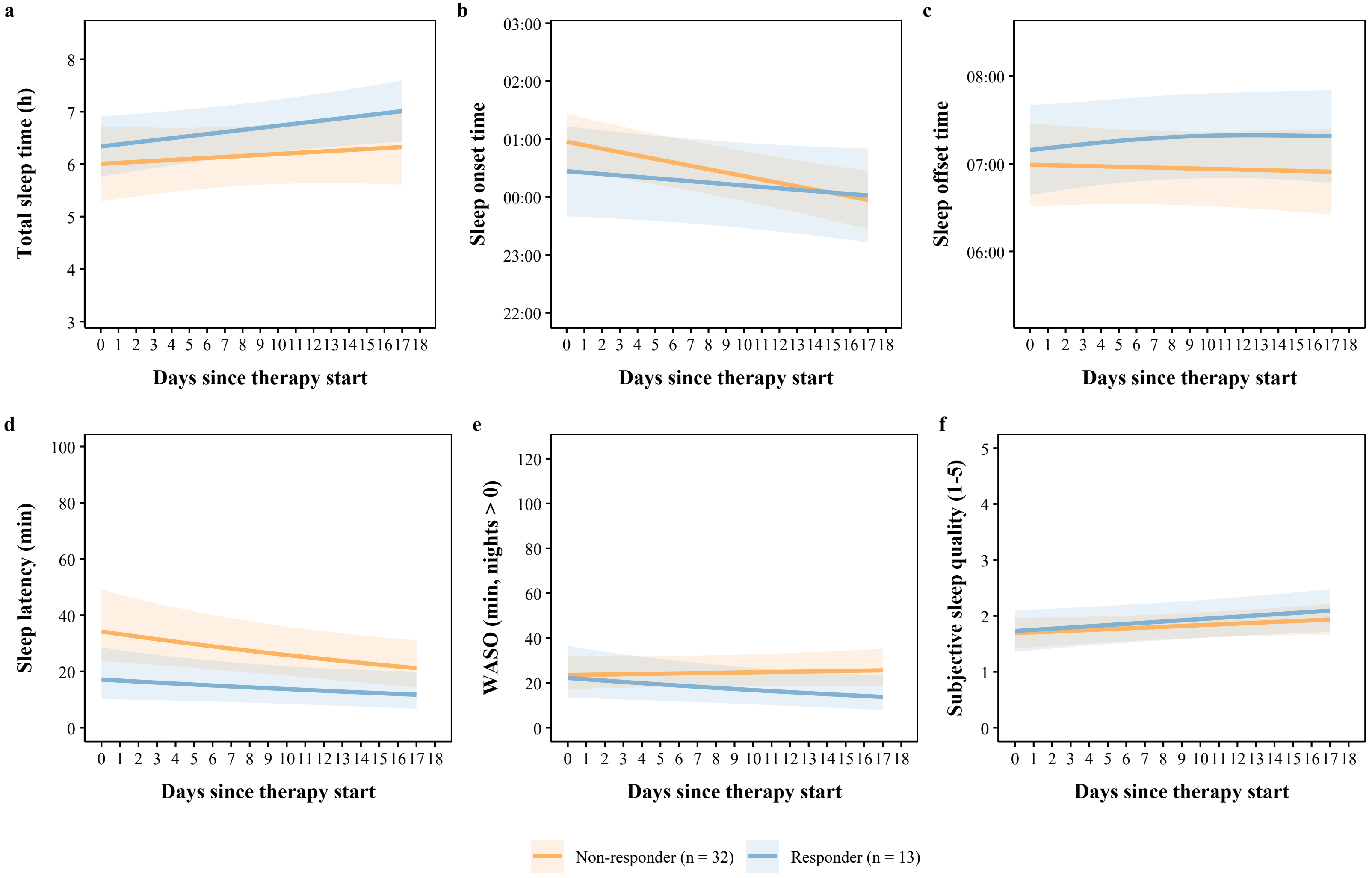

Figure S2. Exploratory subgroup-specific population-level weekday trajectories of sleep variables over time during bright-light therapy, stratified by treatment response status (responders vs. non-responders). Subplots show (a) total sleep time, (b) sleep onset time, (c) sleep offset time, (d) sleep latency, (e) Wake After Sleep Onset (WASO), and (f) subjective sleep quality. Curves were obtained by re-fitting the main GAMM separately within each subgroup and are shown with uncertainty bands. Response was defined as a $\geq$ 50% reduction in QIDS from baseline to week 3. WASO = wake after sleep onset.

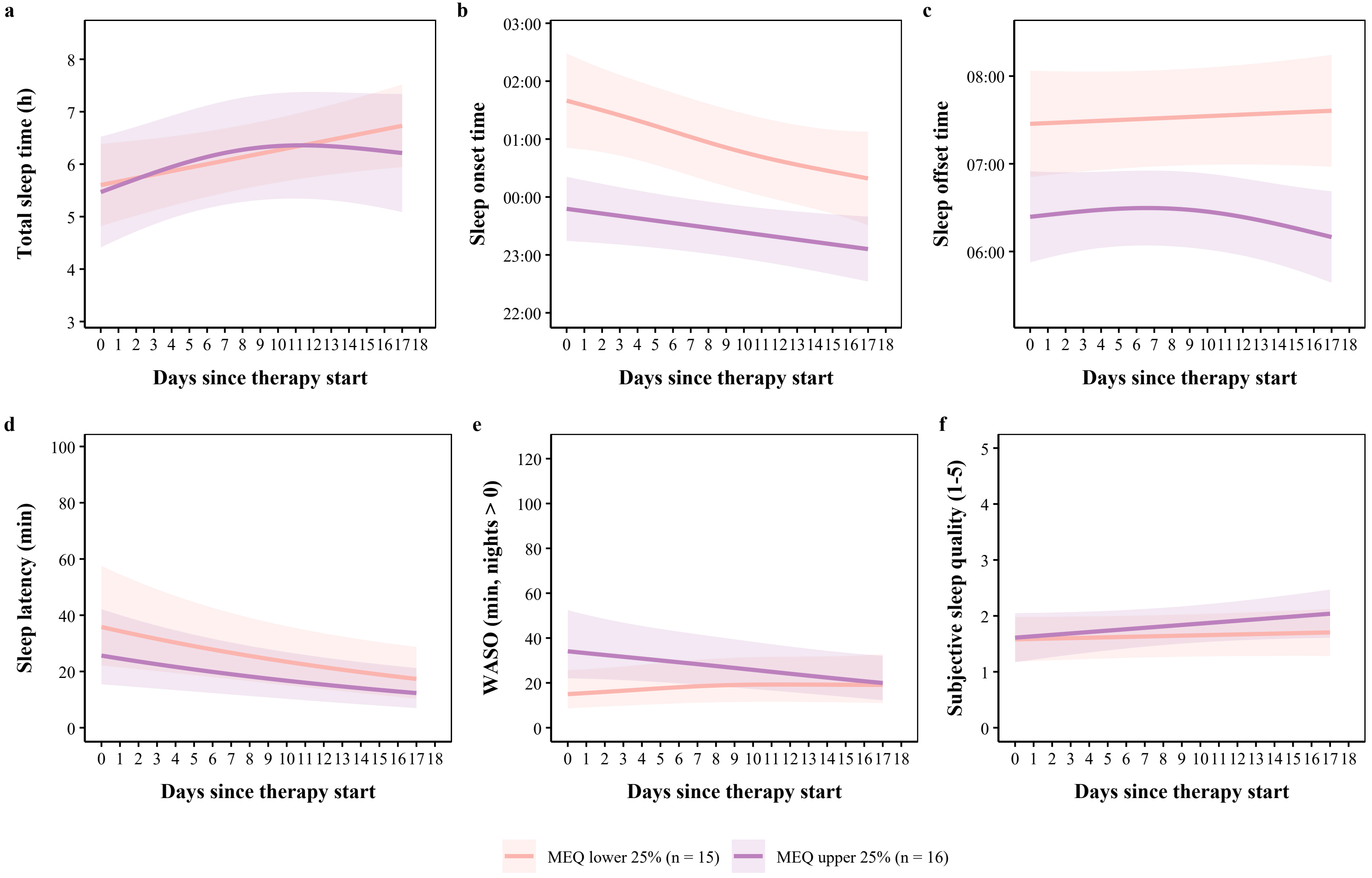

Figure S3. Exploratory subgroup-specific population-level weekday trajectories of sleep variables over time during bright-light therapy, stratified by chronotype. Curves were obtained by re-fitting the main GAMM separately within each subgroup and are shown with uncertainty bands. Chronotype was assessed using the Morningness-Eveningness Questionnaire (MEQ). Subgroups were based on the lower and upper 25% of participants. Subplots show (a) total sleep time, (b) sleep onset time, (c) sleep offset time, (d) sleep latency, (e) Wake After Sleep Onset (WASO), and (f) subjective sleep quality.

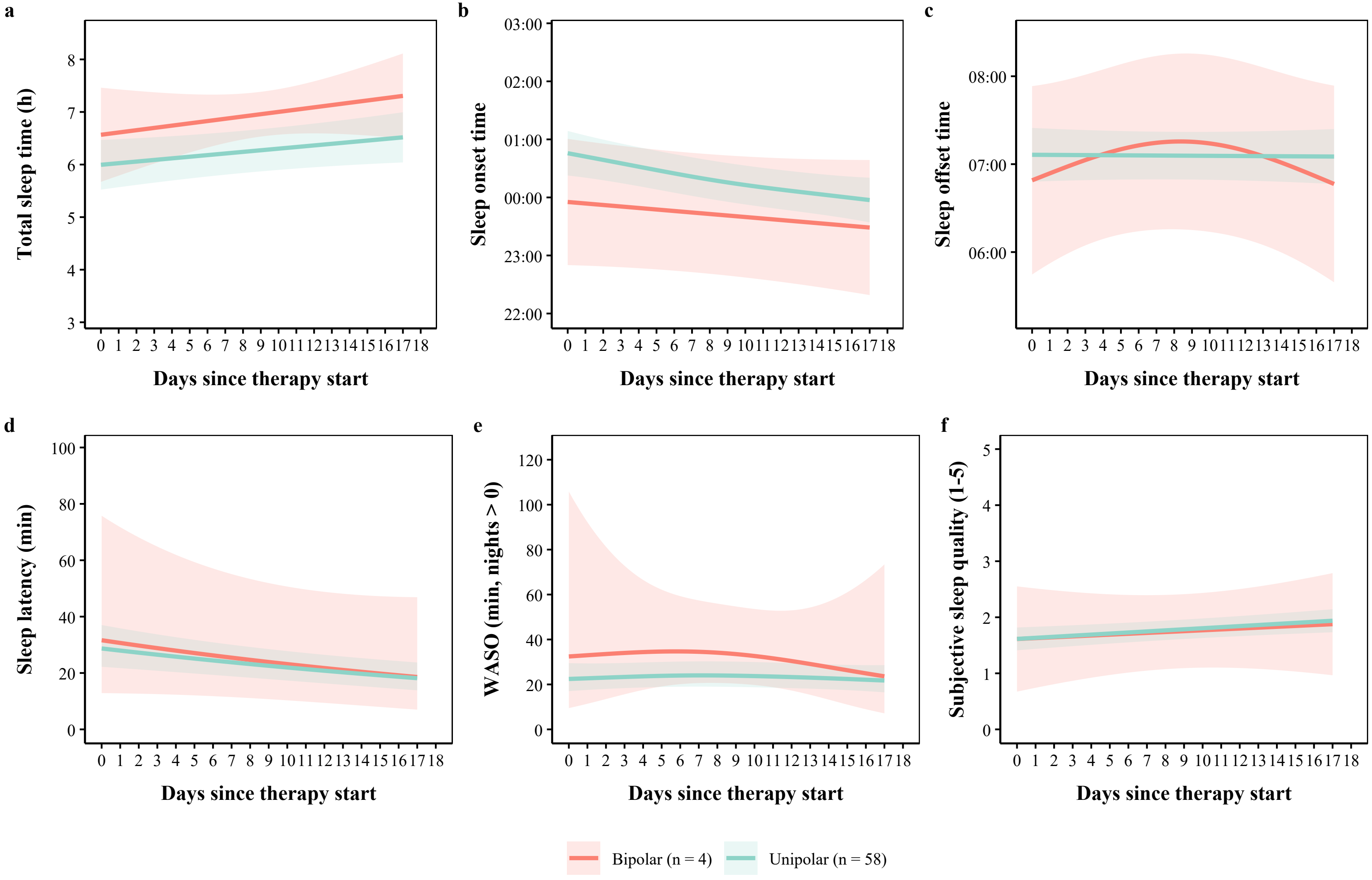

Figure S4. Exploratory subgroup-specific population-level weekday trajectories of sleep variables over time during bright-light therapy, stratified by depression diagnosis (unipolar/bipolar). Subplots show (a) total sleep time, (b) sleep onset time, (c) sleep offset time, (d) sleep latency, (e) Wake After Sleep Onset (WASO), and (f) subjective sleep quality. Curves were obtained by re-fitting the main GAMM separately within each subgroup and are shown with uncertainty bands. For four participants in the total sample, no official diagnosis was available.

S3. Confounder Adjusted GAMM

To examine the robustness of the primary models, all analyses were repeated with adjustment for age, gender, baseline QIDS-SR, baseline PSQI, chronotype (MEQ), and medication use (sleep medication and antidepressants). Full model summaries are presented in Table S2. Overall, the adjusted models were largely consistent with the primary models, with predicted trajectories showing similar directional changes across therapy days.

For sleep onset, the earlier timing observed in the primary models remained statistically significant after adjustment (overall smooth: $F = 19.39$, $p < 01$), and predicted trajectories continued to show a progressive advance in sleep timing. In addition, a weekend-specific smooth reached statistical significance ($F = 5.62$, $p = 18$), whereas the primary model showed only a weekday-specific effect.

For SOL, the primary model showed no significant temporal smooth ($p = .60$), whereas the adjusted model showed a significant overall smooth ($F = 97$, $p = 03$). Predicted trajectories in both specifications were consistent with a gradual decline in SOL across therapy days.

For the probability of experiencing any WASO, the primary model showed a significant overall smooth ($\chi^2 = 8.14$, $p = 20$), whereas the adjusted model showed a significant weekday-specific smooth ($\chi^2 = 84$, $p = 05$) and no significant global effect ($p = .805$). Predicted weekday probabilities remained consistent with a modest decline across therapy days.

For SSQ, weekday- and weekend-specific smooths that were significant in the primary models (weekday $p = 02$, weekend $p = 35$) were no longer statistically significant after adjustment (all $p > .61$), although predicted trajectories in both models continued to show gradual increases across therapy days.

For total sleep time, sleep offset, and WASO duration, temporal smooth terms remained non-significant in both adjusted and unadjusted models, with predicted trajectories showing only minor directional changes across therapy days.

Participant-specific smooth terms remained highly significant across all outcomes in both specifications (all $p < 01$), indicating substantial between-individual variability in sleep trajectories during therapy.

Table S2. Model Fit Indices, Fixed Effects, and Smooth-Term Statistics From Confounder-Adjusted GAMMs Examining Temporal Dynamics of Sleep During Bright-Light Therapy

Outcome	n	EDF_{total}	Adj. R^2	Dev. Expl. (%)	AIC	Fixed effect	Fixed effect stats	Smooth term	edf	Test stat	p
**Total Sleep Time (hours)**	638	74.7	.60	65.1	2235	Weekend	$\beta = 1.25, t = 9.45, p < .001$	s(Days)	1.00	$F = 1.18$	.277
						Age	$\beta = -0.02, t = -1.53, p = .126$	s(Days) : Weekday	0.00027	$F = 0.01$	.998
						Gender (Male)	$\beta = -0.81, t = -1.98, p = .048$	s(Days) : Weekend	1.00	$F = 0.75$	.389
						QIDS baseline	$\beta = -0.06, t = -0.90, p = .368$	s(Days, subjno)	72.69	$F = 4.28$	$< .001$
						PSQI baseline	$\beta = -0.20, t = -3.26, p = .001$				
**Sleep onset (across midnight)**	637	66.1	.58	62.3	1950	Weekend	$\beta = 0.38, t = 3.57, p < .001$	s(Days)	1.00	$F = 19.39$	$< .001$
						Age	$\beta = -0.01, t = -0.61, p = .544$	s(Days) : Weekday	0.00010	$F = 0.00$	.999
						QIDS baseline	$\beta = -0.01, t = -0.19, p = .853$	s(Days) : Weekend	1.00	$F = 5.62$	.018
						PSQI baseline	$\beta = 0.04, t = 0.63, p = .526$	s(Days, subjno)	64.14	$F = 6.75$	$< .001$
**Sleep offset (hours)**	634	85.9	.74	77.7	1533	Weekend	$\beta = 1.43, t = 7.87, p < .001$	s(Days)	1.0031	$F = 0.07$	.793
						Age	$\beta = -0.01, t = -1.18, p = .237$	s(Days) : Weekday	0.5965	$F = 2.05$	.225
						QIDS baseline	$\beta = 0.04, t = 0.74, p = .460$	s(Days) : Weekend	1.5290	$F = 3.80$	.058
						PSQI baseline	$\beta = -0.13, t = -2.68, p = .008$	s(Days, subjno)	82.81	$F = 9.18$	$< .001$
**Sleep latency (log min + 1)**	640	68.0	.67	70.3	1253	Weekend	$\beta = -0.04, t = -0.70, p = .486$	s(Days)	1.00	$F = 9.07$	.003
						Age	$\beta = -0.01, t = -1.70, p = .090$	s(Days) : Weekday	0.00020	$F = 0.00$	.999
						Gender (Male)	$\beta = 0.09, t = 0.36, p = .717$	s(Days) : Weekend	1.00	$F = 0.85$	.358
						QIDS baseline	$\beta = -0.05, t = -1.34, p = .182$	s(Days, subjno)	65.97	$F = 6.95$	$< .001$
						PSQI baseline	$\beta = 0.14, t = 3.91, p < .001$				
**WASO (log min + 1; nights > 0)**	382	57.4	.62	68.1	770	Weekend	$\beta = 0.16, t = 1.73, p = .084$	s(Days)	1.0011	$F = 0.00$	.996
						Age	$\beta = -0.00, t = -0.15, p = .884$	s(Days) : Weekday	0.7313	$F = 3.39$	.100
						QIDS baseline	$\beta = 0.02, t = 0.46, p = .649$	s(Days) : Weekend	1.00	$F = 0.23$	.634
						PSQI baseline	$\beta = 0.08, t = 2.53, p = .012$	s(Days, subjno)	54.66	$F = 4.37$	$< .001$
**Pr(any WASO)**[a]	617	70.1	.62	61.1	475	Weekend	$\beta = -2.97, z = -3.20, p = .001$	s(Days)	1.0004	$\chi^2 = 0.06$	.805
						Age	$\beta = 0.08, z = 2.02, p = .043$	s(Days) : Weekday	0.9079	$\chi^2 = 8.04$	.005
						QIDS baseline	$\beta = 0.24, z = 1.30, p = .194$	s(Days) : Weekend	1.8296	$\chi^2 = 4.07$	.114
						PSQI baseline	$\beta = -0.01, z = -0.04, p = .966$	s(Days, subjno)	66.40	$\chi^2 = 190.39$	$< .001$
**Subjective Sleep Quality**	627	74.6	.45	52.0	1397	Weekend	$\beta = 0.21, t = 3.01, p = .003$	s(Days)	1.0001	$F = 0.26$	.613
						Age	$\beta = -0.00, t = -0.49, p = .626$	s(Days) : Weekday	1.0005	$F = 0.14$	.707
						QIDS baseline	$\beta = -0.02, t = -0.67, p = .506$	s(Days) : Weekend	0.0011	$F = 0.44$	.975
						PSQI baseline	$\beta = -0.06, t = -2.16, p = .031$	s(Days, subjno)	72.63	$F = 3.37$	$< .001$

Note. EDF = effective degrees of freedom. WASO = wake after sleep onset. EDF_{total} reflects the summed effective degrees of freedom across smooth terms. [a] Binomial GAMM with logit link; smooths evaluated using χ^2 tests.

S4. Included vs. Excluded Participants

To evaluate potential selection effects, participants included in the sleep-analysis sample were compared with those excluded from that sample on available demographic and clinical characteristics. Continuous variables were compared using Welch's t-tests. Binary variables were compared using Fisher's exact tests, with odds ratios (ORs) reported as effect-size summaries. Holm correction was applied across all 18 comparisons.

Table S3 summarizes the descriptive statistics and group comparisons. Across the available variables, included and excluded participants were broadly similar. Before correction for multiple testing, the included group showed higher baseline depressive symptom severity, lower chronotype scores, a higher prevalence of depressive disorder, and a higher prevalence of ADHD than the excluded group. However, none of these differences remained statistically significant after Holm correction, although the depressive-disorder contrast approached significance. Overall, these findings suggest only minor differences between included and excluded participants on the variables available for comparison.

Table S3. Comparison of participants included in versus excluded from the sleep-analysis sample

Variable	Included	Excluded	Statistic	p	p_{Holm}
Age (years)	38.5 (13.92)	41.8 (14.63)	$t = -1.50$	.137	1 00
Baseline QIDS-SR	16.7 (4 7)	15.2 (4.66)	$t = 2.30$	23	.398
Baseline PSQI	9.4 (3.48)	9.1 (3.48)	$t = 0.56$	.576	1 00
Chronotype (MEQ total score)	43.5 (12.43)	47.5 (10.40)	$t = -2.13$	36	.543
Mean point reduction	4.40 (4.35)	6.12 (4.61)	$t = -1.79$	76	1 00
Gender (female)	34 (51.5%)	86 (60.6%)	OR = 0.79	.536	1 00
Depressive Disorder	40 (60.6%)	59 (41.5%)	OR = 2.55	04	66
Seasonal Affective Disorder	25 (37.9%)	63 (44.4%)	OR = 0.85	.646	1 00
Bipolar Disorder	4 (6.1%)	22 (15.5%)	OR = 0.38	.108	1 00
Dysthymia	5 (7.6%)	20 (14.1%)	OR = 0.54	.257	1 00
Autism Spectrum Disorder	16 (24.2%)	28 (19.7%)	OR = 1.41	.357	1 00
Personality Disorder	13 (19.7%)	35 (24.6%)	OR = 0.81	.720	1 00
ADHD	12 (18.2%)	11 (7.7%)	OR = 2.84	28	.446
PTSD	7 (10.6%)	18 (12.7%)	OR = 0.88	1 00	1 00
Antidepressant use	30 (45.5%)	65 (45.8%)	OR = 1.11	.762	1 00
Sleep medication use	11 (16.7%)	40 (28.2%)	OR = 0.55	.159	1 00
Remission (QIDS-SR < 6)	8 (17.8%)	14 (32.6%)	OR = 0.45	.142	1 00
Response (50% symptom reduction)	12 (26.7%)	20 (46.5%)	OR = 0.42	76	1 00

Note. Total included sample consisted of 66 participants, but only 45 completed both baseline and end-of-treatment depression ratings. Total excluded participants is 146. Values are mean (SD) for continuous variables and n (%) for binary variables. Continuous variables were compared using Welch's t-tests. Binary variables were compared using Fisher's exact tests, with odds ratios (ORs) shown as effect-size summaries. Holm correction was applied across all 18 comparisons. Seasonal Affective Disorder was defined as meeting at least three of the four DSM-derived seasonal pattern criteria: recurrent winter episodes, remission outside of winter, a pattern persisting for at least two years, and a higher frequency of winter than summer episodes.

S5. GAMM specification and diagnostic checks

This supplement provides the exact generalized additive mixed model (GAMM) specifications and diagnostic summaries used to evaluate whether the fitted models provided an adequate representation of the daily diary data.

Model specification

For continuous outcomes (total sleep time, sleep onset, sleep offset, log-transformed sleep onset latency, log-transformed WASO duration, and subjective sleep quality), GAMMs were fitted in `mgcv` using restricted maximum likelihood (REML). Models included a parametric weekend-versus-weekday effect to capture average level differences, a population-level smooth for therapy day, a day-type-specific smooth deviation allowing weekday and weekend trajectories to differ over time, and a participant-specific factor-smooth for therapy day. In `mgcv` notation, the Gaussian models were specified as:

```
outcome ~ weekend +
  s(DaysSinceStart, k = 6) +
  s(DaysSinceStart, by = weekend, k = 3) +
  s(DaysSinceStart, subjno, bs = "fs", k = 3)
```

WASO occurrence was analyzed analogously using a binomial GAMM with a logit link:

```
WASO_any ~ weekend +
  s(DaysSinceStart, k = 6) +
  s(DaysSinceStart, by = weekend, k = 3) +
  s(DaysSinceStart, subjno, bs = "fs", k = 3)
```

Basis dimensions were intentionally kept modest because the treatment period covered only a short interval of daily observations. Smoothness was estimated by penalization using REML, so the effective degrees of freedom were determined by the data rather than fixed a priori. Missing diary days were not imputed; all GAMMs were therefore estimated using the available post-processed observations after exclusion of implausible values and participant-specific outliers.

Model diagnostiscs

Model adequacy was evaluated using `gam.check()`, with attention to convergence, Hessian definiteness, gradient ranges, model rank, and basis-dimension checks. All models converged fully under the outer Newton optimizer, and all Hessians were positive definite, indicating stable estimation (Table S4). For total sleep time, sleep onset, sleep offset, sleep onset latency, and WASO occurrence, basis-dimension checks did not suggest that the selected values of `k` were too low: k-indices were close to 1 and associated p-values were non-significant (Table S5). By contrast, the diagnostics for log-transformed WASO duration and subjective sleep quality were somewhat more borderline, with lower k-indices (approximately 0.93–0.94) and several significant p-values in the basis-dimension checks. These findings suggest that the chosen basis dimensions may have been somewhat restrictive for these outcomes, and therefore their smooth estimates should be interpreted somewhat more cautiously than those for the other sleep outcomes.

Visual inspection of the diagnostic plots indicated some tail deviations in the QQ plots for several outcomes, particularly for total sleep time, sleep onset, sleep offset, sleep onset latency, and log-transformed WASO duration, but no residual patterns suggesting substantial model misspecification or deviations severe enough to compromise interpretation of the reported temporal trends.

Table S4. GAMM convergence and fit diagnostics

Outcome	Family	n	Rank	Iterations	Hessian	Gradient range
Total sleep time	Gaussian	959	208/209	9	Positive definite	[−00033, 00049]
Sleep onset	Gaussian	954	208/209	8	Positive definite	[−00117, 00298]
Sleep offset	Gaussian	949	208/209	7	Positive definite	[−00020, 00007]
Sleep latency	Gaussian	955	208/209	11	Positive definite	[−00009, 00021]
WASO duration	Gaussian	619	196/197	9	Positive definite	[−00063, 00121]
Subjective sleep quality	Gaussian	941	208/209	10	Positive definite	[−00043, 00091]
WASO occurrence	Binomial	933	205/206	6	Positive definite	[−00018, 00012]

Note. All models were fitted by REML using the outer Newton optimizer. Gradient ranges are reported from `gam.check()`.

Table S5. Basis-dimension diagnostics from `gam.check()`

Outcome	Smooth term	k'	EDF	k-index	p
Total sleep time	s(DaysSinceStart)	5 0	1 0	1 0	.46
Total sleep time	s(DaysSinceStart) : weekday	2 0	1 0	1 0	.52
Total sleep time	s(DaysSinceStart) : weekend	2 0	0 0	1 0	.52
Total sleep time	s(DaysSinceStart, subjno)	198 0	123.10	1 0	.44
Sleep onset	s(DaysSinceStart)	5 0	0 1	0.99	.34
Sleep onset	s(DaysSinceStart) : weekday	2 0	1.47	0.99	.34
Sleep onset	s(DaysSinceStart) : weekend	2 0	1 0	0.99	.38
Sleep onset	s(DaysSinceStart, subjno)	198 0	124.92	0.99	.34
Sleep offset	s(DaysSinceStart)	5 0	1 0	0.98	.33
Sleep offset	s(DaysSinceStart) : weekday	2 0	1 0	0.98	.26
Sleep offset	s(DaysSinceStart) : weekend	2 0	0 0	0.98	.32
Sleep offset	s(DaysSinceStart, subjno)	198 0	138 0	0.98	.30
Sleep latency	s(DaysSinceStart)	5 0	1 0	0.99	.34
Sleep latency	s(DaysSinceStart) : weekday	2 0	1 0	0.99	.35
Sleep latency	s(DaysSinceStart) : weekend	2 0	0 0	0.99	.36
Sleep latency	s(DaysSinceStart, subjno)	198 0	99.92	0.99	.37
WASO duration	s(DaysSinceStart)	5 0	1 0	0.94	50
WASO duration	s(DaysSinceStart) : weekday	2 0	1.63	0.94	70
WASO duration	s(DaysSinceStart) : weekend	2 0	0 0	0.94	45
WASO duration	s(DaysSinceStart, subjno)	186 0	104.20	0.94	45
Subjective sleep quality	s(DaysSinceStart)	5 0	0 0	0.93	05
Subjective sleep quality	s(DaysSinceStart) : weekday	2 0	1 0	0.93	10
Subjective sleep quality	s(DaysSinceStart) : weekend	2 0	1 0	0.93	10
Subjective sleep quality	s(DaysSinceStart, subjno)	198 0	110.74	0.93	05
WASO occurrence	s(DaysSinceStart)	5 0	1.75	0.97	.26
WASO occurrence	s(DaysSinceStart) : weekday	2 0	1 0	0.97	.30
WASO occurrence	s(DaysSinceStart) : weekend	2 0	1 0	0.97	.26
WASO occurrence	s(DaysSinceStart, subjno)	195 0	94 7	0.97	.23

Note. Low p-values for the k-index check may indicate that the basis dimension was too low, particularly when EDF approaches k'. Values shown correspond to the first `gam.check()` output for each model.
